# Attenuation of palmitic acid-induced lysyl oxidase overexpression in the ovary contributes to the improvement of ovulation in obesity by metformin

**DOI:** 10.1093/hropen/hoae002

**Published:** 2024-01-10

**Authors:** Chuyue Zhang, Wang-Sheng Wang, Guangxin Yao, Yanan Zhu, Yikai Lin, Jiangwen Lu, Kang Sun, Yun Sun

**Affiliations:** Center for Reproductive Medicine, Ren Ji Hospital, School of Medicine, Shanghai Jiao Tong University, Shanghai, China; Shanghai Key Laboratory for Assisted Reproduction and Reproductive Genetics, Shanghai, China; Center for Reproductive Medicine, Ren Ji Hospital, School of Medicine, Shanghai Jiao Tong University, Shanghai, China; Shanghai Key Laboratory for Assisted Reproduction and Reproductive Genetics, Shanghai, China; Center for Reproductive Medicine, Ren Ji Hospital, School of Medicine, Shanghai Jiao Tong University, Shanghai, China; Shanghai Key Laboratory for Assisted Reproduction and Reproductive Genetics, Shanghai, China; Xiangyang Central Hospital, Affiliated Hospital of Hubei University of Arts and Science, Hubei, China; Center for Reproductive Medicine, Ren Ji Hospital, School of Medicine, Shanghai Jiao Tong University, Shanghai, China; Shanghai Key Laboratory for Assisted Reproduction and Reproductive Genetics, Shanghai, China; Center for Reproductive Medicine, Ren Ji Hospital, School of Medicine, Shanghai Jiao Tong University, Shanghai, China; Shanghai Key Laboratory for Assisted Reproduction and Reproductive Genetics, Shanghai, China; Center for Reproductive Medicine, Ren Ji Hospital, School of Medicine, Shanghai Jiao Tong University, Shanghai, China; Shanghai Key Laboratory for Assisted Reproduction and Reproductive Genetics, Shanghai, China; Center for Reproductive Medicine, Ren Ji Hospital, School of Medicine, Shanghai Jiao Tong University, Shanghai, China; Shanghai Key Laboratory for Assisted Reproduction and Reproductive Genetics, Shanghai, China

**Keywords:** obesity, ovulation, lysyl oxidase, extracellular matrix, palmitic acid, metformin

## Abstract

**STUDY QUESTION:**

Does palmitic acid (PA), the most common saturated free fatty acid (FFA) in individuals with obesity, contribute to anovulation through upregulation of the collagen-crosslinking enzyme lysyl oxidase (LOX) in the ovary?

**SUMMARY ANSWER:**

Increased PA in individuals with obesity can cause LOX upregulation via the activation of hypoxia-inducible factor-1α (HIF-1α), resulting in abnormal collagen deposition in the ovary and anovulation, which can be ameliorated by metformin therapy.

**WHAT IS KNOWN ALREADY:**

The underlying cause of anovulation in individuals with obesity is poorly defined, and accumulating evidence indicates that hormonal disturbance, insulin resistance, and inflammation may all play a role in the development of ovulation disorders in individuals with obesity. However, it remains to be determined whether PA plays a role in the regulation of LOX expression, thus disrupting ovarian extracellular matrix (ECM) remodelling in the ovary and resulting in impaired ovulation in individuals with obesity.

**STUDY DESIGN, SIZE, DURATION:**

PA concentration and LOX protein abundance and activity in follicular fluid and ovarian tissue were compared between control (n = 21) subjects, patients with obesity with ovulation (n = 22), and patients with obesity with anovulation (n = 16). The effect of PA on LOX protein expression, and the underlying mechanism, was examined in primary human granulosa cells *in vitro*. The improvements in obesity conditions induced by LOX inhibition combined with metformin were investigated in a high-fat diet-induced obese rat model.

**PARTICIPANTS/MATERIALS, SETTING, METHODS:**

The abundance of PA concentration and LOX activity was measured via a LOX activity assay and ELISA, respectively. The effect of PA on LOX protein expression was examined in the presence or absence of inhibitors of signalling molecules and siRNA-mediated knockdown of the putative transcription factor. Chromatin immunoprecipitation assays were subsequently conducted to further identify the responsible transcription factor. The role of metformin in the treatment of anovulation by LOX inhibition was investigated in a high-fat diet (HFD)-induced obese rat model. The numbers of retrieved total oocytes and metaphase II oocytes were recorded upon ovarian stimulation. Masson’s trichrome staining was used to measure the total collagen content, and immunohistochemical staining and western blotting were used to measure LOX, HIF-1α, and collagen I and IV in the ovary.

**MAIN RESULTS AND THE ROLE OF CHANCE:**

Significantly increased FFA, LOX, and collagen abundance were observed in the ovaries of obese women with anovulation, compared to healthy controls or obese women with ovulation. In a HFD-induced obese rat model, metformin corrected the distortion of ovarian morphology by decreasing LOX and collagen protein abundance in the ovary and improving oestrous cyclicity and ovulation. PA increased LOX expression via the activation of HIF-1α in human granulosa cells, which was attenuated by metformin.

**LARGE SCALE DATA:**

N/A.

**LIMITATIONS, REASONS FOR CAUTION:**

Several other saturated and polyunsaturated FFAs, such as stearic acid and arachidonic acid, are also increased in the blood of individuals with obesity, and increased levels of other FFAs may also contribute to the development of anovulation in individuals with obesity, which needs to be further verified in the future.

**WIDER IMPLICATIONS OF THE FINDINGS:**

Elevated PA in individuals with obesity can cause LOX dysregulation via activation of HIF-1α, resulting in abnormal collagen deposition in the ovary and anovulation. This dysregulation can be ameliorated by metformin therapy through its local effect on ECM remodelling in the ovary, which is independent of its systemic effect on insulin sensitivity and chronic inflammation.

**STUDY FUNDING/COMPETING INTEREST(S):**

This work was supported by the National Natural Science Foundation of China (grant numbers 82101730, 82130046, and 31900598) and Innovative Research Team of High-level local Universities in Shanghai (SHSMU-ZLCX20210201). All the authors declare no conflicts of interest in relation to this work.

WHAT DOES THIS MEAN FOR PATIENTS? Obesity in women is linked with ovulation disorders. The development of follicles (which contain the eggs) in ovaries relies on degradation of the extracellular matrix (a supportive network, which contains the structural protein collagen) accompanied by the downregulation of lysyl oxidase (LOX), a collagen cross-linking enzyme. In this study, we observed that the amount of LOX and collagen increases in the ovaries of obese women who do not ovulate. Furthermore, we demonstrated that palmitic acid (PA), the most common saturated free fatty acid (FFA), could increase LOX through the activation of a specific factor (hypoxia-inducible factor 1-α) in human granulosa cells (cells that are critical for ovarian function, including oestrogen synthesis and helping egg growth), which was inhibited by metformin (commonly prescribed to treat lack of ovulation caused by insulin resistance in infertile women). Metformin administration also improved ovulation in obese women (and in an experimental rat model of obesity) by reducing the amount of LOX and collagen in the ovary. To summarize, we showed that in obesity, PA increases LOX expression and excessive accumulation of collagen in the ovary, which may restrict follicle growth, resulting in impaired ovulation and thereby leading to infertility. Metformin improved ovulatory functions in obese individuals by reducing these effects of PA. Therefore, our findings may prompt clinicians to address the dysregulation of FFAs and provide new evidence that supports the therapeutic use of metformin in the treatment of ovulation disorders in obese women.

## Introduction

Obesity is a pandemic issue (Loret de Mola, 2009). In many parts of the world, obesity is one of the greatest threats to public health (Talmor and Dunphy, 2015), as obesity is linked to conditions such as dyslipidaemia, hypertension, type 2 diabetes, coronary heart disease, stroke, and even cancer (Hensrud and Klein, 2006). Compelling evidence indicates that obesity is also inextricably linked with infertility, particularly in women (Castillo-Martinez *et al.*, 2003; Bhattacharya *et al.*, 2009). Infertility in obese women is largely attributed to menstrual irregularities, poor gamete development, impaired uterine receptivity, and ovulation disorders (Pasquali *et al.*, 2007; Loret de Mola, 2009; Koning *et al.*, 2010; Dağ and Dilbaz, 2015; Fichman *et al.*, 2020). The deleterious effects of ovulation disorders, either irregular ovulation or anovulation, are particularly worthy of attention since ovulation disorders not only are a common cause of female infertility (Weiss and Clapauch, 2014) but also may impact women’s health in the long term via alteration of the endocrine milieu, leading to increased risks of endometrial and breast cancers (Sadia *et al.*, 2009). Therefore, elucidating the mechanism underlying ovulation disorders in obese individuals is of paramount importance for ensuring the well-being of women.

Accumulated evidence indicates that hormonal disturbance, insulin resistance, and inflammation may all play a role in the development of ovulation disorders in individuals with obesity (Gosman *et al.*, 2006; Bohler *et al.*, 2010; Jungheim *et al.*, 2012). Increased levels of saturated free fatty acids (FFAs) in the serum have been shown to be linked to the development of all the pathological processes described above in individuals with obesity (Boden, 1997, 2008). Palmitic acid (PA), the most common saturated free FFA (Carta *et al.*, 2017), has been shown to be an important contributor to the development of insulin resistance as well as low-grade inflammation in a variety of tissues, including the ovary, in individuals with obesity (Boden, 1997; Yang *et al.*, 2012; Xu *et al.*, 2019; Zhou *et al.*, 2020). Interestingly, PA has also been shown to be involved in the development of fibrosis in nonovarian tissues, and upregulation of lysyl oxidase (LOX) expression is one of the fundamental underlying mechanisms (Li *et al.*, 2016; Chu *et al.*, 2019). LOX is a copper-dependent amine oxidase (Erler *et al.*, 2009) that catalyses the crosslinking of collagen fibrils. Crosslinked collagen fibrils not only confer strength but also provide resistance to degradation by proteases (Vater *et al.*, 1979).

Normal ovarian follicle development and ovulation require finely coordinated interactions between the follicle and extracellular matrix (ECM) remodelling (Richards *et al.*, 1998; Woodruff and Shea, 2007; Young and McNeilly, 2010). Ovarian ECM remodelling of the follicular basement membrane and thecal stroma (Auersperg *et al.*, 1994; Huet *et al.*, 1997; Rodgers *et al.*, 2000) is needed not only for proper endocrine functions of follicular cells but also for follicle growth and ovulation (Richards *et al.*, 1998; Harlow *et al.*, 2003; Lind *et al.*, 2006; Woodruff and Shea, 2007; Young and McNeilly, 2010). Decrosslinking of collagen fibrils through downregulation of LOX expression is needed for the normal development of the follicle and ovulation (Harlow *et al.*, 2003), whereas excessively crosslinked collagen fibrils can restrict follicle growth and impair ovulation (Umehara *et al.*, 2022). However, it remains to be determined whether PA may play a role in the regulation of LOX expression, thus disrupting ovarian ECM remodelling and resulting in impaired ovulation in individuals with obesity.

Metformin is commonly prescribed to treat anovulation associated with insulin resistance in infertile women (Practice Committee of the American Society for Reproductive Medicine, 2017; Vial *et al.*, 2019). Observational studies indicate that metformin can lower serum androgen levels and restore menstrual cyclicity (Wisniewski and Petersen, 2009). However, no mechanistic studies have been performed to validate the therapeutic use of metformin for the treatment of anovulation in individuals with obesity. Notably, metformin has been shown to suppress collagen deposition in lung fibrosis and in the ovaries of postmenopausal women (Rangarajan *et al.*, 2018; McCloskey *et al.*, 2020). Thus, we postulated that metformin might correct PA-induced abnormal ovarian ECM remodelling through downregulation of LOX expression, thereby improving ovulation in individuals with obesity. Herein, we addressed these hypotheses by using human ovarian tissue and granulosa cells obtained from women with obesity as well as from an obese rat model.

## Materials and methods

### Participant recruitment

All procedures for the human studies were performed following the Declaration of Helsinki and approved by the Ethics Committee of Ren Ji Hospital, School of Medicine, Shanghai Jiao Tong University (KY2021-230-B), and all the participants provided written informed consent to participate in the study. Based on Chinese demographic features, the obesity diagnosis in this study was in accordance with the following criteria: normal range, 18.50 < BMI < 24.00 kg/m^2^; overweight, 24.00 ≤ BMI < 28.00 kg/m^2^; and obese, BMI ≥ 28.00 kg/m^2^ (Zhou, 2002). Infertile women with a normal BMI and with only tubal factor or male factor infertility were recruited into the control lean group (n = 38). Women with obesity (BMI ≥ 28.00 kg/m^2^) with a normal ovarian reserve were subdivided into two groups: obese with regular ovulation and a cycle (n = 22) and obese with anovulation and an irregular cycle (n = 16). The latter group excluded patients with PCOS or other diseases that might cause ovulation disorders. To evaluate the basic endocrine profile and ovarian reserve of the recruited women, we determined basal serum hormonal profiles, including FSH, LH, testosterone (T), estradiol (E2), and anti-Müllerian hormone (AMH) levels, using chemiluminescence assay kits (DxI 800; Beckman Access Health Co., Brea, CA, USA). To evaluate the lipid profile of the recruited women, we determined triglyceride (TG) levels using an enzymatic method (Hitachi Modular P Chemistry Analyzer, Hitachi, Ltd, Tokyo, Japan). For evaluation of the insulin sensitivity status of the recruited women, fasting blood glucose was measured using an enzymatic colorimetric glucose oxidase method with a commercial kit (Cobas 600; Roche, Basel, Switzerland), and plasma insulin levels were measured with a chemiluminescence assay kit (Beckman Access Health Co.). The homeostasis model of assessment for insulin resistance (HOMA-IR) was calculated with the following equation: fasting serum insulin [μIU/ml] × fasting serum glucose [mmol/L]/22.5.

For determination of whether metformin treatment can improve ovulation, 57 women who were obese but did not have PCOS who underwent ovulation induction therapy at our reproductive center during the last 5 years were recruited and subdivided into two groups: the letrozole-only group (n = 34) and the letrozole plus metformin group (n = 23). Letrozole is a widely prescribed aromatase inhibitor in ovulation induction therapy (Casper and Mitwally, 2011). Letrozole was given on the fifth day of a spontaneous or progestin withdrawal cycle, at 2.5 mg daily for 5 days in both groups. In the letrozole plus metformin group, metformin was administered at least 1 month before the first cycle of ovulation induction at a dose of 500 mg daily. Ultrasound and serum progesterone (P_4_) level (>5 ng/ml) were used to confirm ovulation during the treatment cycle.

### Immunohistochemical and Masson’s trichrome staining of human ovaries

For analysis of the distribution pattern of collagen and associated factors in the ovaries of lean and obese women with or without anovulation, human ovarian tissue was collected from patients who underwent laparoscopic surgery to remove benign cystic teratomas of the ovary during the follicular phase before ART. A total of 11 patients were recruited and further divided into lean (n = 5), obese with ovulation (n = 3), and obese with anovulation (n = 3) groups. Immunohistochemical staining of ovarian tissue sections was performed using an antibody against LOX raised in rabbit (1:100; Abcam, Cambridge, UK). Non-immune rabbit IgG (Proteintech, Wuhan, China) served as a negative control. After washing, the sections were sequentially incubated with the corresponding biotinylated secondary antibody raised in horse and avidin–biotin complex conjugated with horseradish peroxidase (Vector Laboratories, Burlingame, CA, USA). The horseradish peroxidase activity was subsequently developed as a red colour using 3-amino-9-ethyl carbazole (Vector Laboratories). The sections were semiquantified with Image-Pro Plus 6.0 software (Media Cybernetics, Rockville, MD, USA), and the average optical density of the positive spots in 10 visual fields of each section at 200× magnification was analysed.

The collagen volume fraction (CVF) was determined by analysing ovarian sections stained with Masson’s trichrome as described previously (Lu *et al.*, 2012). The Masson’s trichrome-stained (blue) and nonstained areas of the entire section, excluding the follicular cavity and vasculature, were determined using ImageJ software 6.0 with a colour-based threshold (Goto, 2019). The CVF was calculated by dividing the blue-stained area by the total area of the ovarian section (exclusive of follicular cavity and vasculature).

### Collection of human follicular fluid and culture of primary granulosa and KGN cells

Follicular fluid and ovarian granulosa cells were collected from recruited women who underwent IVF following oocyte retrieval. Ovarian stimulation was performed under the GnRH antagonist protocol for all patients. After adequate follicle development, hCG (Livzon Pharmaceutical Group Co., Zhuhai, China) was administered to trigger follicular maturation. Thirty-six hours later, oocyte retrieval was performed, and follicular fluid was collected from size-matched dominant follicles (18–20 mm), which were pooled. Granulosa-lutein cells in the collected follicular fluid were recovered by centrifugation at 2500*g* for 15 min at 37°C, resuspended in culture medium (DMEM/F12; Thermo Fisher Scientific, Waltham, MA, USA) and dispersed in 0.1% hyaluronidase (Sigma, Burlington, MA, USA) at 37°C for 10 min. The dispersed granulosa cell suspension was subjected to Ficoll-Paque (GE-Health Care Bio-Science, Uppsala, Sweden) density centrifugation for further purification. After centrifugation, the purified granulosa cells were either stored at −80°C for subsequent protein extraction for analysis via western blotting or resuspended in DMEM/F12 with 10% foetal bovine serum (FBS) (Thermo Fisher Scientific). The follicular fluid without cells was stored at −80°C for later determination of the FFA concentration (Bhat *et al.*, 2022; Dai *et al.*, 2022) and LOX activity (Ash *et al.*, 2021; Das *et al.*, 2022) with commercial assay kits (FFAs: Abcam; LOX: AAT Bioquest, Pleasanton, CA, USA) following the manufacturers’ instructions. KGN cells (a gift from Shangdong University), a human granulosa-like tumor cell line, were cultured in phenol red-free DMEM/F12 supplemented with 10% FBS and 1% antibiotics.

### Obese rat model

All animal experimentation procedures were conducted following the accepted regulation of animal care and were approved by the Institutional Review Board of Ren Ji Hospital, School of Medicine, Shanghai Jiao Tong University. An obese rat model (n = 25) was generated by feeding 6-week-old female Sprague–Dawley rats (Jiesijie Laboratory Animal, Shanghai, China) a high-fat diet (HFD) containing 10% lard, 10% yolk, 1% cholesterol, 0.2% cholate and 78.8% standard diet for 16 weeks, and the control lean rats (n = 25) were fed a standard diet. Metformin (200 mg/kg) therapy was started by gavage daily in half of both the lean and obese rats by the 13th week for 4 consecutive weeks, and the other half received water by gavage as a control. Body weight was recorded every week. On the 16th week, data on the body and visceral fat pad weights, blood lipid and hormonal profiles, and glucose tolerance test were collected from the obese rats treated with or without metformin therapy. Ovulation status was examined via observation of the oestrous cycle and via a superovulation test.

The oestrous cycle was assessed by examining the vaginal cytology for 10 consecutive days before death. A superovulation test was conducted on some of the rats in the 16th week by injection (i.p.) of pregnant mare’s serum gonadotrophin (300 IU/kg) followed by hCG injection (300 IU/kg) (Ningbo Sansheng Pharmaceutical Co., Ningbo, China) 48 h later. Cumulus–oocyte complexes were collected from the oviduct 16 h after hCG injection. Oocytes were freed from cumulus cells by digestion with 0.1% hyaluronidase (Vitrolife, Frölunda, Sweden) for 5 min. Images were taken using a Zeiss microscope, and the number of oocytes that reached metaphase II (MII) was recorded.

A glucose tolerance test was conducted by i.p. injection of d-glucose (2.0 g/kg body weight) after the animals had fasted for 16 hours (17:00–09:00) but had free access to drinking water. Blood was taken through the tail vein, and plasma glucose levels before and 15, 30, 60, 90, and 120 min after i.p. glucose injection were measured with an Accu-Chek glucose monitor (Roche, Indianapolis, IN, USA).

Under avertin anaesthesia, blood was collected via cardiac exsanguination to determine the lipid profile, after which the perigonadal and retroperitoneal fat pads and ovaries were dissected and weighed to calculate the ratio of perigonadal or retroperitoneal fat pad weight (g)/100 g of body weight. The blood lipid profile was examined by measuring FFAs (Abcam), TGs, and total cholesterol (including high- and low-density lipoprotein: HDL and LDL) with ELISA kits (Abcam). The blood hormonal profile was also examined by measuring FSH, LH, E_2_, and P_4_ with ELISA kits (Nan Jing Jian Cheng Bioengineering Institute, Jiangsu, China), which were used together with ovarian histological examination, oestrous cycle observation, and a superovulation test to evaluate ovulation status. For analysis of the accumulation of fat droplets, freshly sliced sections of ovarian tissues were incubated in 60% isopropanol for 3 min and subsequently stained with Oil Red O reagent for 10 min. The slices were washed with 60% isopropanol and water and subsequently stained with haematoxylin (Gao *et al.*, 2021). For histological examination of ovarian cystic formation and collagen deposition, the dissected ovary was fixed in 4% paraformaldehyde, embedded in paraffin, and sections cut through the maximal axis at a thickness of 5 μm. Sections were stained with haematoxylin and eosin (HE) for observation of cystic formation or with Masson’s trichrome for observation of collagen deposition. Immunohistochemical staining was performed to evaluate the distribution of LOX and hypoxia-inducible factor-1α (HIF-1α) (1:100; Cell Signaling Technology, Danvers, MA, USA). The abundance of LOX and collagen in the ovary was also determined via western blotting as described below.

### Treatment of primary human granulosa and KGN cells *in vitro*

Human granulosa cells were cultured for 3 days in DMEM/F12 supplemented with 10% FBS before reagent treatment in the same culture medium without phenol red or FBS. The cells were treated with PA diluted with anhydrous ethanol (30 and 300 μM, 24 h) (Sigma, Burlington, MA, USA) to examine any concentration-dependent effect on LOX and HIF-1α expression in serum-free medium, and an equal volume of ethanol solution was added to the vehicle control group. The cells were also treated with PA (300 μM, 24 h) to observe HIF-1α nuclear translocation. For analysis of the role of HIF-1α in the effect of PA on LOX expression, the cells were treated with PA (300 μM, 24 h) in the presence or absence of BAY 87-2243 (10 μM, 24 h; Selleck, Shanghai, China), an inhibitor of HIF-1α (Wu *et al.*, 2018), or by siRNA-mediated knockdown of HIF-1α. The effect of metformin on PA-induced LOX expression and HIF-1α nuclear translocation was examined by treating the cells with PA (300 μM, 24 h) in the presence or absence of metformin (1 mM, Sigma–Aldrich, St Louis, MI, USA).

For siRNA transfection, siRNA against HIF-1α (5′-GAAAGUCAUGAACCACGUUTT-3′) (GenePharma Co., Shanghai, China) was transfected into granulosa cells via electroporation. Briefly, granulosa cells were mixed with 50 nM siRNA in Opti-MEM (Life Technologies, Inc., Grand Island, NY, USA) immediately after isolation via electroporation at 175 V for 5 ms using a NEPA21 electroporator (Nepa Gene Co., Ltd, Chiba, Japan). Randomly scrambled siRNA served as a negative control. After dilution with DMEM/F12 supplemented with 10% charcoal-stripped FBS, the granulosa cells were transferred to culture plates and incubated for 72 h before PA treatment.

### Protein extraction for analysis via Western blotting

After reagent treatment, granulosa cells were lysed in ice-cold radioimmunoprecipitation assay (RIPA) lysis buffer (Active Motif, Carlsbad, CA, USA) containing inhibitors of both protease (Sigma) and phosphatase (Active Motif). After centrifugation at 15 000*g*, the supernatant containing total cellular protein was collected to measure the abundance of LOX, HIF-1α, collagen I, and collagen IV. For analysis of HIF-1α nuclear translocation upon PA treatment, nuclear and cytoplasmic proteins were extracted from the cells using a nuclear extraction kit (Active Motif). After the protein concentration was determined via the Bradford assay (Beyotime, Hangzhou, China), 50 μg of protein from each sample was electrophoresed on a 10% sodium dodecyl sulphate (SDS)-polyacrylamide gel and transferred to nitrocellulose. This blot was then blocked with nonfat milk followed by incubation with antibodies against LOX (Abcam), HIF-1α (Cell Signaling Technology), collagen I (Proteintech, Wuhan, China) or collagen IV (Proteintech) at 1:1000 dilutions overnight at 4°C. After washing, corresponding secondary antibodies (Proteintech) conjugated with horseradish peroxidase were applied. The peroxidase activity band was developed with a chemiluminescence detection system (Millipore, Darmstadt, Germany) and visualized using a G-Box chemiluminescence image capture system (Syngene, Cambridge, UK). Internal loading controls were generated by probing the same blot with antibodies against β-actin (1:1000; Proteintech) and GAPDH (1:1000; Proteintech) for total cellular protein or with an antibody against Lamin A/C (1:1000; Cell Signaling) for nuclear protein.

### Chromatin immunoprecipitation

Chromatin immunoprecipitation (ChIP) was performed to examine the binding of HIF-1α to the *LOX* promoter after treatment with PA (300 μM, 12 h). The granulosa cells were fixed with 1% formaldehyde. After termination of treatment with glycine, the cells were lysed with 1% SDS supplemented with a protease inhibitor cocktail. The lysed cells were sonicated to shear the chromatin DNA. After precleaning, the sheared DNA was immunoprecipitated with an antibody against HIF-1α. Equal amounts of preimmune IgG served as the negative control. The immunoprecipitate was then incubated with Magna ChIP Protein A + G Magnetic Beads (Millipore) and pulled down using a magnetic stand. After reverse crosslinking and digestion with ribonuclease A/proteinase K, the sheared DNA was extracted and analysed via quantitative real-time PCR (qRT–PCR) with the following primers amplifying the putative HIF-1α-binding site at the *LOX* promoter: 5′-GTTCGCCCCAGATTAAGCCA-3′ (forward) and 5′-CGATTGGAAACGTGCAAGGC-3′ (reverse). The same amount of sheared DNA without antibody precipitation served as the input control. The ratio of DNA precipitated by the HIF-1α antibody to that precipitated by the input control was obtained to indicate the amount of transcription factor bound to the *LOX* promoter.

### Statistical analysis

All the data are reported as the mean ± SEM. Data were analyzed with GraphPad Prism software (version 9.0, Boston, MA, USA). The Kolmogorov–Smirnov test was used to assess whether the continuous variables were normally distributed. For normally distributed data, paired or unpaired Student’s t-test or one-way ANOVA tests followed by Student–Newman–Keuls multiple comparisons tests were used where appropriate to assess significant differences. The ovulatory status of women in the letrozole-only and letrozole plus metformin treatment groups was tabulated, and the outcomes were compared with the χ^2^ test. *P* < 0.05 indicated statistical significance.

## Results

### Clinical and biochemical features of the recruited participants

The clinical and biochemical features of the recruited participants are displayed in Table 1. There were no significant differences in age; endocrine profiles, including FSH, E2, or T basal levels; or AMH among the three groups (control lean women, obese women with ovulation, and obese women with anovulation). However, fasting insulin and glucose levels, HOMA-IR, and TG levels were significantly greater, and basal LH levels were significantly lower in the obese women than in the control lean women. BMI and cycle length were significantly greater but the number of oocytes retrieved was significantly lower in the obese women with anovulation than in the obese women with ovulation or in the control lean women. Notably, both FFA levels and LOX activity were significantly greater in the follicular fluid of the obese women with or without anovulation than in that of the control lean women but were greatest in the obese women with anovulation. These features indicated that insulin resistance and dyslipidaemia were present in obese women either with or without anovulation, but those with obesity with anovulation appear to have a significantly greater BMI as well as greater FFA levels and LOX activity in the follicular fluid.

**Table 1.** Demographic features of recruited subjects in a study of anovulation in obesity.

Clinical parameters	Control	Obese with ovulation	Obese with anovulation
N	21	22	16
Age (years)	28.19 ± 0.78	29.45 ± 0.67	30.00 ± 0.67
BMI (kg/m^2^)	20.28 ± 0.35	30.88 ± 0.44	33.44 ± 0.87
Cycle length (days)	30.00 ± 0.54	35.32 ± 1.61	46.06 ± 4.82***^,^^#^
Basal FSH (mIU/ml)	6.95 ± 0.37	6.45 ± 0.31	6.47 ± 0.35
Basal LH (mIU/ml)	5.69 ± 0.46	4.14 ± 0.38*	3.99 ± 0.28*
Basal E_2_ (pg/ml)	36.67 ± 3.56	34.57 ± 3.12	36.54 ± 3.70
Basal T (nmol/l)	0.73 ± 0.07	0.73 ± 0.07	0.77 ± 0.10
AMH (ng/ml)	3.93 ± 0.34	3.73 ± 0.30	3.64 ± 0.27
No. oocytes retrieved	14.24 ± 1.50	11.05 ± 0.84	9.44 ± 0.67*
Fasting glucose (mmol/ml)	5.09 ± 0.08	5.58 ± 0.14*	5.67 ± 0.21*
Fasting insulin (IU/ml)	7.22 ± 0.22	11.91 ± 0.73***	12.25 ± 0.84***
HOMA-IR	1.63 ± 0.04	3.00 ± 0.24***	3.14 ± 0.30***
Triglycerides (mmol/ml)	1.18 ± 0.08	1.73 ± 0.11***	2.04 ± 0.11***
FFAs in FF (μM)	46.37 ± 1.57	55.04 ± 1.85*	66.73 ± 3.39***^,^^##^
LOX activity in FF (Fluorescence units × 100/μl)	79.10 ± 4.84	108.05 ± 5.89**	133.70 ± 7.54***^,^^#^

AMH: anti-Müllerian hormone; E_2_: estradial; FF: follicular fluid; FFAs: free fatty acids; HOMA-IR: homeostasis model of assessment for insulin resistance; LOX: lysyl oxidase; T: testosterone.

Women with PCOS were not included in the study. Data are mean  ±  SEM values. Statistical analysis was performed with one-way ANOVA tests followed by Student–Newman–Keuls multiple comparisons tests.

* *P *<* *0.05 vs control.

** *P *<* *0.01 vs control.

*** *P *<* *0.001 vs control.

^#^ *P *<* *0.05 vs obesity with ovulation.

^##^ *P *<* *0.01 vs obesity with ovulation.

### Histological examination of the ovaries of obese and control lean women

**Figure 1.**
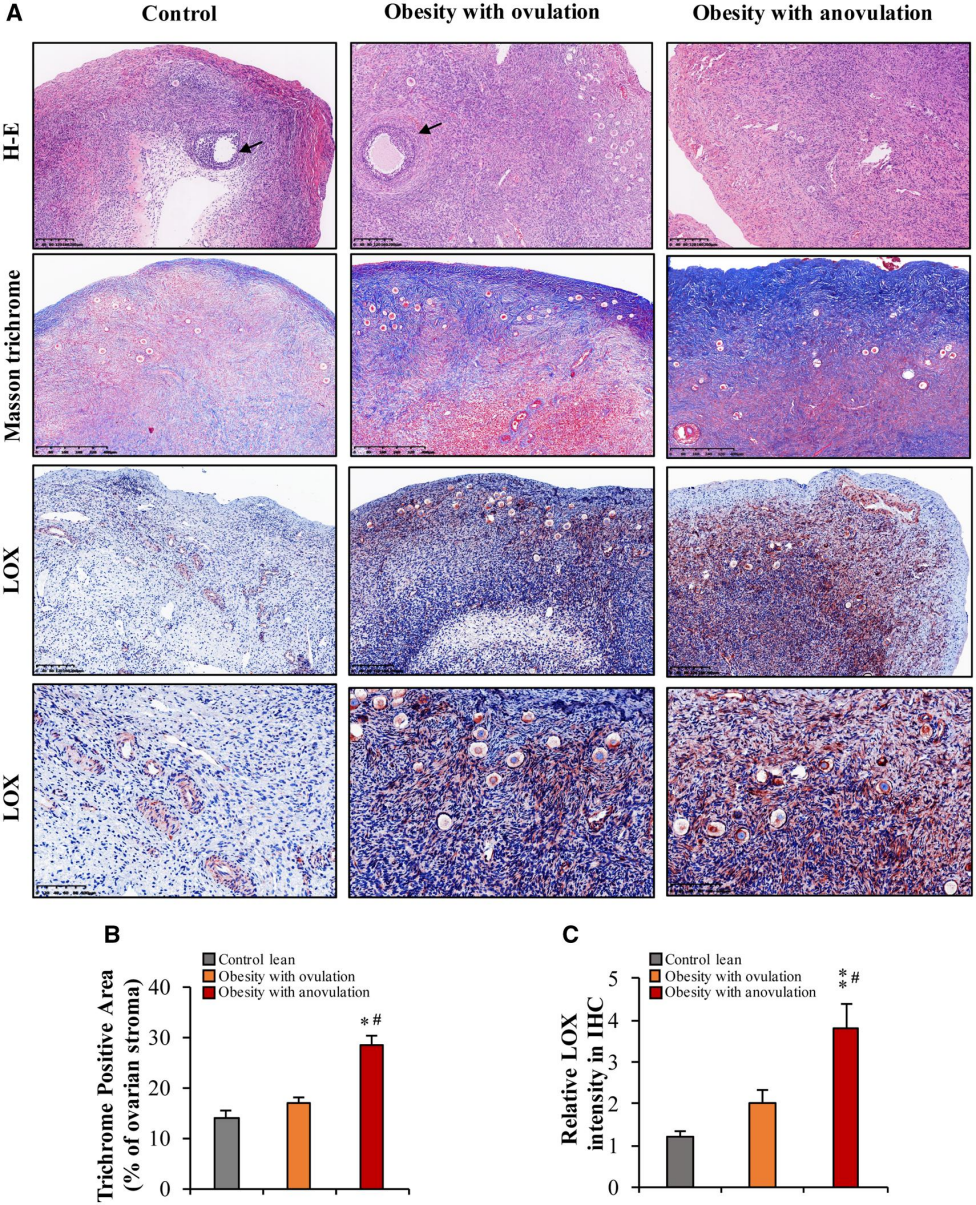
**Excessive collagen deposition and lysyl oxidase overexpression in the ovaries of obese women with anovulation.** (**A**) Haematoxylin and eosin staining showing a few antral follicles (arrow) in the ovarian cortex of the lean control group and the obese with ovulation group but not in the obese with anovulation group. (**A** and **B**) Masson’s staining showing the collagen (blue) distribution pattern in the ovarian cortex in the lean control, obese with ovulation, and obese with anovulation groups. (**A** and **C**) Immunohistochemical staining of LOX in the ovaries of the lean control, obese with ovulation, and obese with anovulation groups. Images are representative of 3–5 patients in each group. The data are presented as the mean ± SEM. Statistical analysis was performed with one-way ANOVA tests followed by Student–Newman–Keuls multiple comparisons tests. *P < 0.05, **P < 0.01 vs the control group; ^#^*P* < 0.05 vs the obesity with ovulation group. H–E: haematoxylin and eosin; IHC: immunohistochemistry; LOX: lysyl oxidase.

Staining with HE revealed a few antral follicles in the ovaries of both the control lean and obese women with ovulation but not in the ovaries of the obese women with anovulation (Fig. 1A). Masson’s trichrome staining revealed increased collagen abundance (blue) in the stroma surrounding the follicles and beneath the ovarian epithelial surface (Fig. 1A). The ovarian CVF was significantly greater in the obese women with anovulation than in the obese women with ovulation or in the control lean women (Fig. 1A and B). Immunohistochemical staining revealed more intense LOX staining in ovarian granulosa cells and stroma in the obese women with anovulation than in either the control lean women or the obese women with ovulation (Fig. 1A and C). These results indicate that LOX abundance and collagen deposition are increased in the ovaries of obese women with anovulation.

### Amelioration of ovulation disorder in obese women and in HFD-induced obese rats by metformin

A retrospective study of 57 obese women who received letrozole-only or letrozole plus metformin treatment showed that there were no significant differences in age, BMI or cycle length between the two groups in the first treatment cycle of ovulation induction therapy (Table 2). However, at least one ovulation occurred during 20 out of 34 cycles (59%) in the letrozole-only group and during 20 out of 23 cycles (87%) in the letrozole plus metformin group (*P *<* *0.05) during the therapy cycle (Table 2), suggesting that metformin can further improve ovulation in obese women.

**Table 2.** Comparison of demographic features and therapeutic effects between the letrozole-only and letrozole plus metformin groups in obese women.

Clinical parameters	Letrozole only (n = 34)	Letrozole plus metformin (n = 23)
Age (years)	32.97 ± 0.41	30.61 ± 0.68
BMI (kg/m^2^)	30.02 ± 0.28	29.24 ± 0.34
Cycle length (days)	34.06 ± 1.31	35.07 ± 1.96
Cycles with ovulation (%)	20 (59%)	20 (87%)*

Data are mean ± SEM. Statistical analysis was performed with unpaired Student’s *t*-test (age, BMI, and cycle length) or χ^2^ test (cycles with ovulation).

* *P *<* *0.05 vs letrozole-only group.

**Figure 2.**
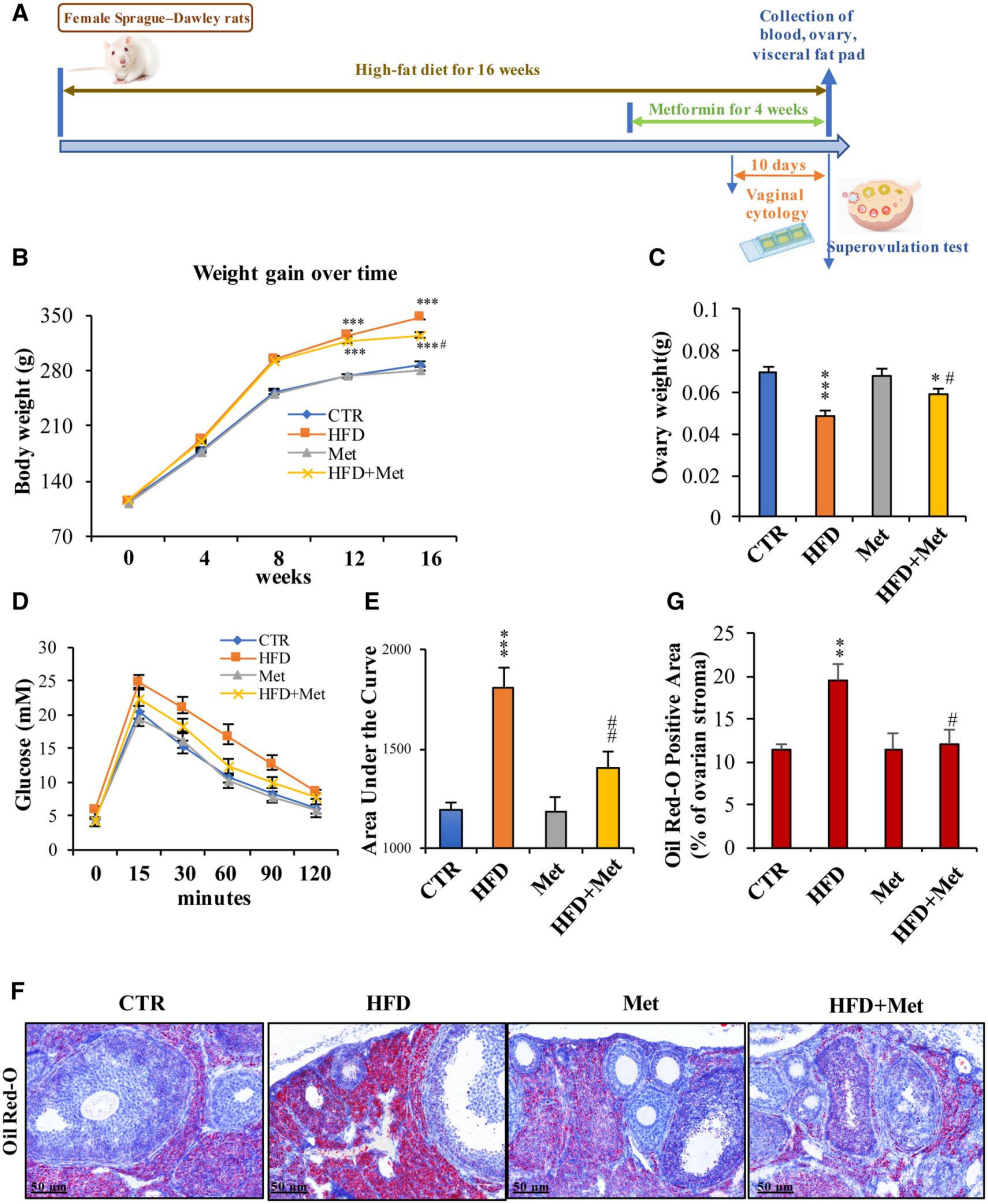
**The therapeutic effect of metformin in a rat obesity model.** (**A**) The timeline of animal experimentation. (**B**) Effect of metformin (Met) on body weight in high-fat diet (HFD)-induced obese rats. (**C**) Effect of metformin on ovarian weight in HFD-induced obese rats. (**D** and **E**) Reversal of HFD-induced glucose intolerance by metformin. (**F** and **G**) Oil Red O staining showed attenuation of lipid accumulation in ovarian tissue induced by metformin in the HFD-induced obese rats (n = 5 for each group). The data are presented as the mean ± SEM. Statistical analysis was performed with one-way ANOVA tests followed by Student–Newman–Keuls multiple comparisons tests. *P < 0.05, **P < 0.01, ***P < 0.001 vs the control group; ^#^*P* < 0.05, ^##^*P* < 0.01 vs the HFD group. CTR: control.

To further investigate the role of metformin in the improvement of ovulation in obesity, we established a HFD-induced obese rat model. The timeline of the animal experiments is illustrated in Fig. 2A. Compared with the control lean rats, the female rats fed an HFD presented significantly greater body weights and percentages of perigonadal and retroperitoneal fat pad weights/body weight, despite the lack of difference in nasoanal length (Fig. 2B and Table 3). The ovarian weight of the HFD group was significantly lower than that of the control group (Fig. 2C). Oil Red O staining revealed strong lipid accumulation in the ovaries of HFD-fed rats (Fig. 2F and G). Increased FFA, TG, and LDL levels; decreased HDL levels; and increased AUC were observed in the HFD group (Table 3 and Fig. 2D and E). All these abnormalities were significantly ameliorated by metformin treatment (Fig. 2B–G and Table 3).

**Table 3.** Naso-anal length, body weight, and retroperitoneal, perigonadal fat pad weights and plasma lipid in a rat obesity model.

	CTR (n = 15)	HFD (n = 10)	Met (n = 15)	HFD + Met (n = 10)
Naso-anal length (cm)	19.81 ± 0.13	20.10 ± 0.13	19.65 ± 0.14	19.9 ± 0.12
Body weight (g)	286.01 ± 5.58	345.89 ± 7.70*	279.98 ± 3.45	323.01 ± 5.92*^,^^#^
Perigonadal fat (g/100 g of body weight)	2.47 ± 0.11	6.05 ± 0.16*	2.13 ± 0.12	5.03 ± 0.20*^,^^#^
Retroperitoneal fat (g/100 g of body weight)	0.50 ± 0.03	2.20 ± 0.13*	0.43 ± 0.03	1.78 ± 0.12*^,^^#^
FFAs (μM)	28.66 ± 2.39	56.21 ± 3.31*	29.92 ± 2.36	44.29 ± 1.70*^,^^#^
Triglycerides (mmol/l)	0.24 ± 0.02	0.42 ± 0.03*	0.21 ± 0.02	0.33 ± 0.02*^,^^#^
Total cholesterol (mmol/l)	1.33 ± 0.03	1.45 ± 0.04	1.32 ± 0.03	1.36 ± 0.03
HDL (mmol/l)	1.89 ± 0.17	1.19 ± 0.11*	1.94 ± 0.18	1.74 ± 0.12^#^
LDL (mmol/l)	0.14 ± 0.01	0.18 ± 0.01*	0.13 ± 0.02	0.15 ± 0.02^#^

CTR: control; FFA: free fatty acids; HDL: high-density lipoprotein; HFD: high fat diet; Met: metformin; LDL: low-density lipoprotein. Data are mean ± SEM. Statistical analysis was performed with one-way ANOVA tests followed by Student–Newman–Keuls multiple comparisons tests.

* *P *<* *0.05 vs CTR.

^#^ *P *<* *0.05 vs HFD.

**Figure 3.**
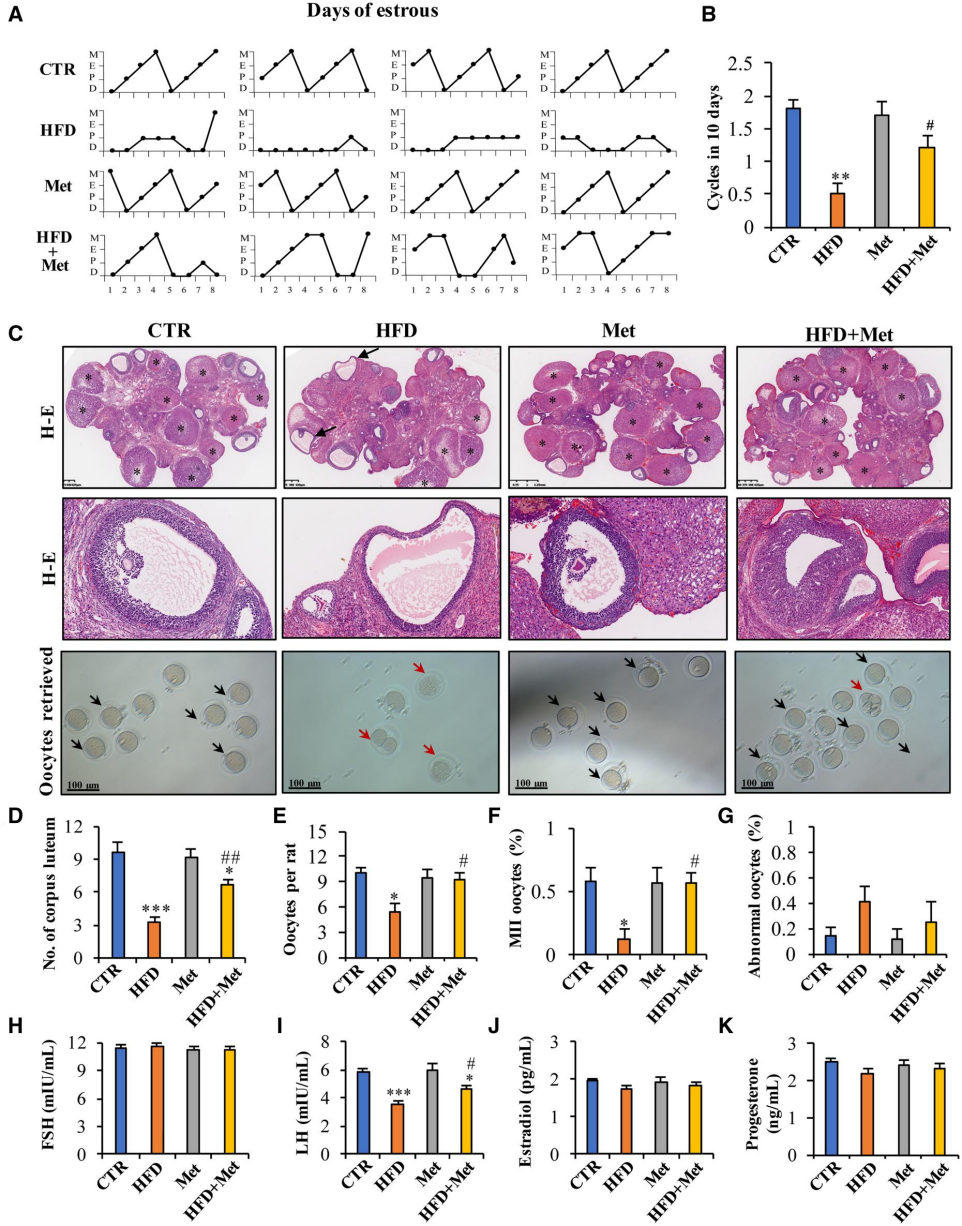
**Improvement of oestrous cyclicity, ovarian morphology, and ovulation by metformin in a rat obesity model.** (**A** and **B**) Effects of metformin (Met) on the oestrous cycle and the number of cycles completed in 10 days. (**C** and **D**) Haematoxylin–eosin staining showed multiple cystic follicles with thinner granulosa cells (arrow) in the high-fat diet (HFD) group, but few cystic follicles were observed in the HFD+metformin group. More corpora lutea (CL, asterisk) were also observed in the HFD group treated with metformin than in the HFD group not treated with metformin. Images are representative of 10 or 15 animals in each group. C is the representative image and D is the average data. (**C** and **E**–**G**) Effect of HFD feeding with or without metformin treatment on the number of oocytes retrieved, MII oocytes, and abnormal oocytes after ovarian superstimulation. The black arrows indicate MII oocytes, and the red arrows indicate abnormal oocytes (n = 5 for each group). C is the representative image and D is the average data. (**H**–**K**) Effect of HFD feeding with or without metformin treatment on the plasma concentrations of FSH, LH, estradiol, and progesterone (n = 10 or 15 for each group). The data are presented as the mean ± SEM. Statistical analysis was performed with one-way ANOVA tests followed by Student–Newman–Keuls multiple comparisons tests. *P < 0.05, ***P < 0.001 vs the CTR (control) group; ^#^*P* < 0.05, ^##^*P* < 0.01 vs the HFD group. D: di-estrus; E: estrus; H–E: haematoxylin and eosin; M, met-estrus; MII: metaphase II; P: pro-estrus.

Metformin administration also improved oestrous cyclicity in the HFD group (Fig. 3A). The number of oestrous cycles completed in 10 days increased by an average of 0.7 cycles in the metformin treatment group compared to the HFD group without metformin treatment (Fig. 3B). Histological examination of the ovaries revealed a few mature follicles with normal layers of granulosa cells, and a significantly greater number of corpora lutea was observed in the ovaries of the HFD-fed rats treated with metformin than in those of the HFD-fed rats not treated with metformin (Fig. 3C and D). Ovarian stimulation showed that metformin treatment increased the number of retrieved total and MII oocytes per rat but had no effect on the number of abnormal oocytes in HFD-fed rats (Fig. 3C and E–G). Moreover, metformin administration elevated plasma LH levels (Fig. 3H–K). These results indicate that metformin can ameliorate ovulation disorders in an obese rat model.

### Attenuation of HFD-induced increases in LOX abundance and collagen deposition in the ovary by metformin in the obese rat model

**Figure 4.**
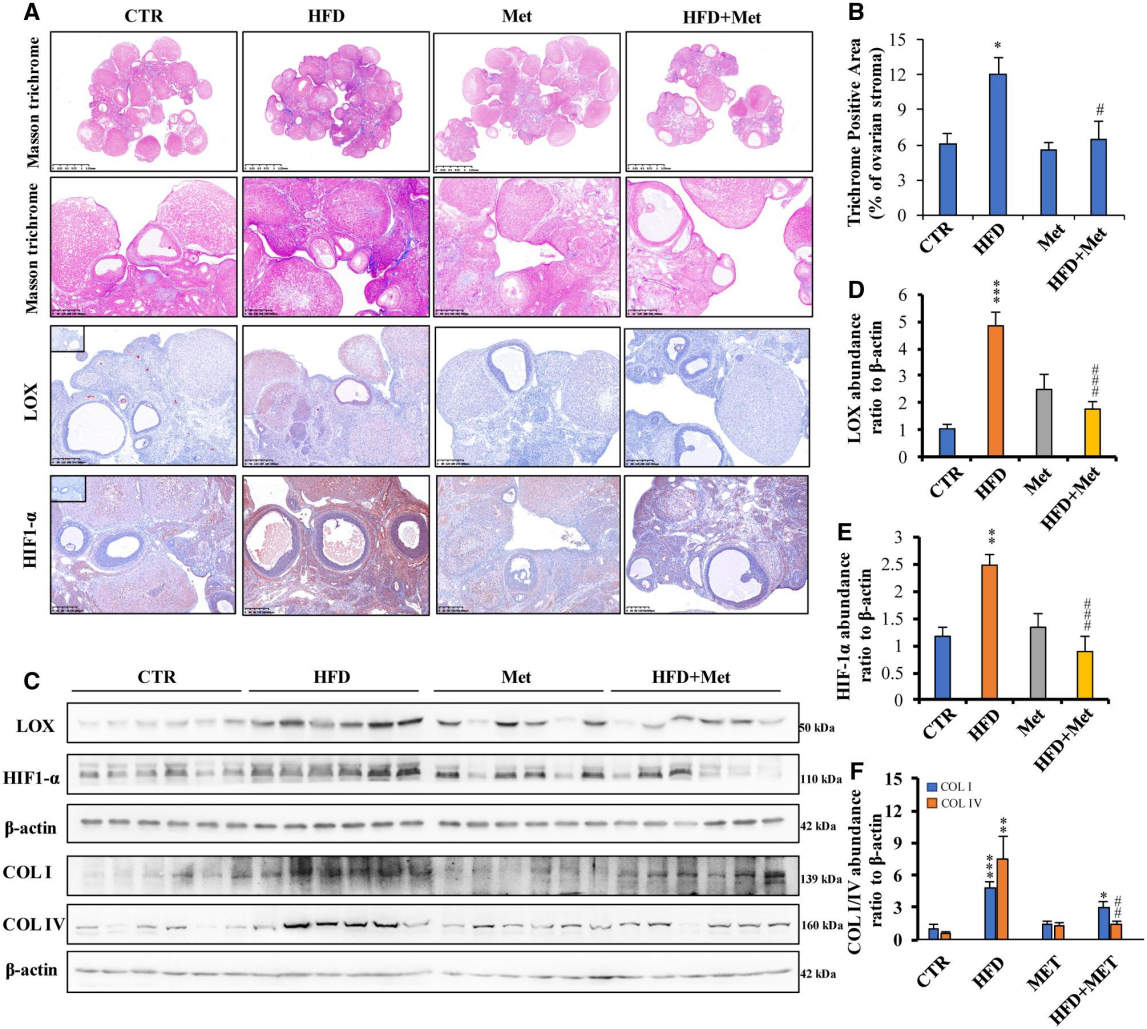
**Amelioration of excessive collagen deposition and lysyl oxidase and hypoxia-inducible factor-1α overexpression by metformin in a rat obesity model.** (**A** and **B**) Masson’s staining showing the distribution of collagen (blue) in the ovary (n = 5 for each group). Immunohistochemical staining of LOX and HIF-1α in the ovary (n = 5 for each group). (**C**–**F**) Western blotting showed LOX, HIF-1α, collagen I (COL I), and collagen IV (COL IV) protein abundance in the ovaries (n = 6 for each group). The data are presented as the mean ± SEM. Statistical analysis was performed with one-way ANOVA tests followed by Student–Newman–Keuls multiple comparisons tests. *P < 0.05, **P < 0.01, ***P < 0.001 vs the control group; ^#^*P* < 0.05, ^##^*P* < 0.01, ^###^*P* < 0.001 vs the HFD group. CTR: control; HFD: high-fat diet; HIF-1α: hypoxia-inducible factor-1α; LOX: lysyl oxidase; Met: metformin.

Masson’s staining revealed increased collagen deposition in the stroma surrounding the follicles and beneath the ovarian epithelial surface with increased CVF in the HFD group, which was significantly attenuated by metformin administration (Fig. 4A and B). Immunohistochemical staining and western blotting showed that the abundance of LOX and HIF-1α significantly increased in the ovaries of the HFD group, which was ameliorated by metformin administration (Fig. 4A and C–E). Western blotting also showed that the abundance of collagen I and collagen IV significantly increased in the ovaries of the HFD group, and metformin administration significantly attenuated the increase in collagen IV abundance, accompanied by a non-significant trend towards a decrease in collagen I abundance (Fig. 4C and F).

### Induction of LOX expression by PA via HIF-1α in human granulosa cells

**Figure 5.**
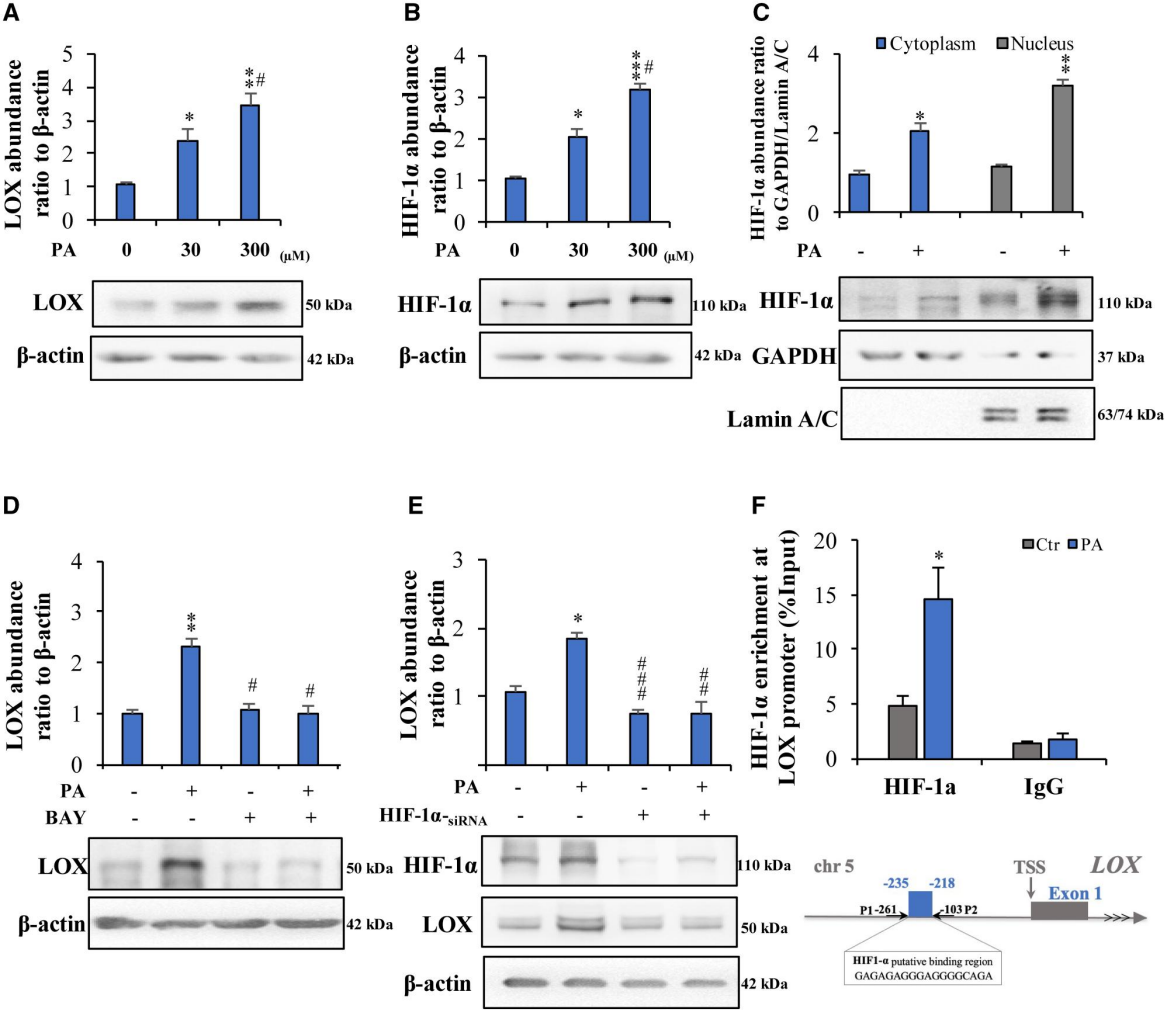
**Induction of lysyl oxidase expression by palmitic acid through hypoxia-inducible factor-1α in primary human granulosa cells *in vitro*.** (**A** and **B**) Concentration-dependent upregulation of LOX and HIF-1α expression by palmitic acid (PA) (0, 30, 300 μM, 24 h) in human ovarian granulosa cells. (**C**) PA (300 μM, 24 h) induced HIF-1α nuclear translocation in human ovarian granulosa cells. (**D** and **E**) Effect of PA (300 μM, 24 h) on the protein abundance of LOX in the presence or absence of an inhibitor of HIF-1α (BAY 87-2243, 10 μM) and siRNA-mediated knockdown of HIF-1α in human ovarian granulosa cells. (**F**) The diagram shows the putative binding sites of HIF-1α in the *LOX* promoter region. The arrows between P1 and P2 indicate the primer alignment positions in the ChIP assay used in this study. TSS, transcription start site. ChIP assays detected HIF-1α enrichment at the binding site of the *LOX* promoter in response to PA (300 μM, 12 h) treatment in human ovarian granulosa cells. IgG served as the negative control. The data are presented as the mean ± SEM of three or four experiments. *P < 0.05, **P < 0.01, ***P < 0.001 vs the control group; ^#^*P* < 0.05, ^##^*P* < 0.01, ^###^*P* < 0.001 vs the PA-treated group. Statistical analysis was performed with one-way ANOVA tests followed by Student–Newman–Keuls multiple comparisons tests (A–E) or paired Student’s *t*-test (F). ChIP: chromatin immunoprecipitation; HIF-1α: hypoxia-inducible factor-1α; LOX: lysyl oxidase; TSS: transcription start site

Treatment with PA (0, 30, 300 μM, 24 h) increased LOX and HIF-1α abundance in a concentration-dependent manner in cultured human granulosa cells (Fig. 5A and B) as well as KGN cells without lipotoxicity or inducing granulosa cell apoptosis (Supplementary Figs S1 and S2). Moreover, PA (300 μM, 24 h) increased the nuclear translocation of HIF-1α in granulosa cells (Fig. 5C). The induction of LOX expression by PA (300 μM, 24 h) was blocked by BAY 87-2243 (10 μM), an inhibitor of HIF-1α (Fig. 5D), or siRNA-mediated knockdown of HIF-1α in granulosa cells (Fig. 5E). Bioinformatics analysis of the *LOX* promoter using hTFtarget software (Zhang *et al.*, 2020) revealed binding sites for HIF-1α spanning the region between −235 and −218 bp (Fig. 5F). ChIP assays showed that PA increased the enrichment of HIF-1α at the binding site region of the *LOX* promoter (Fig. 5F). These results indicate that PA induces LOX expression through the activation of HIF-1α in human granulosa cells.

### Metformin-induced attenuation of PA-induced LOX expression in human granulosa cells *in vitro*

**Figure 6.**
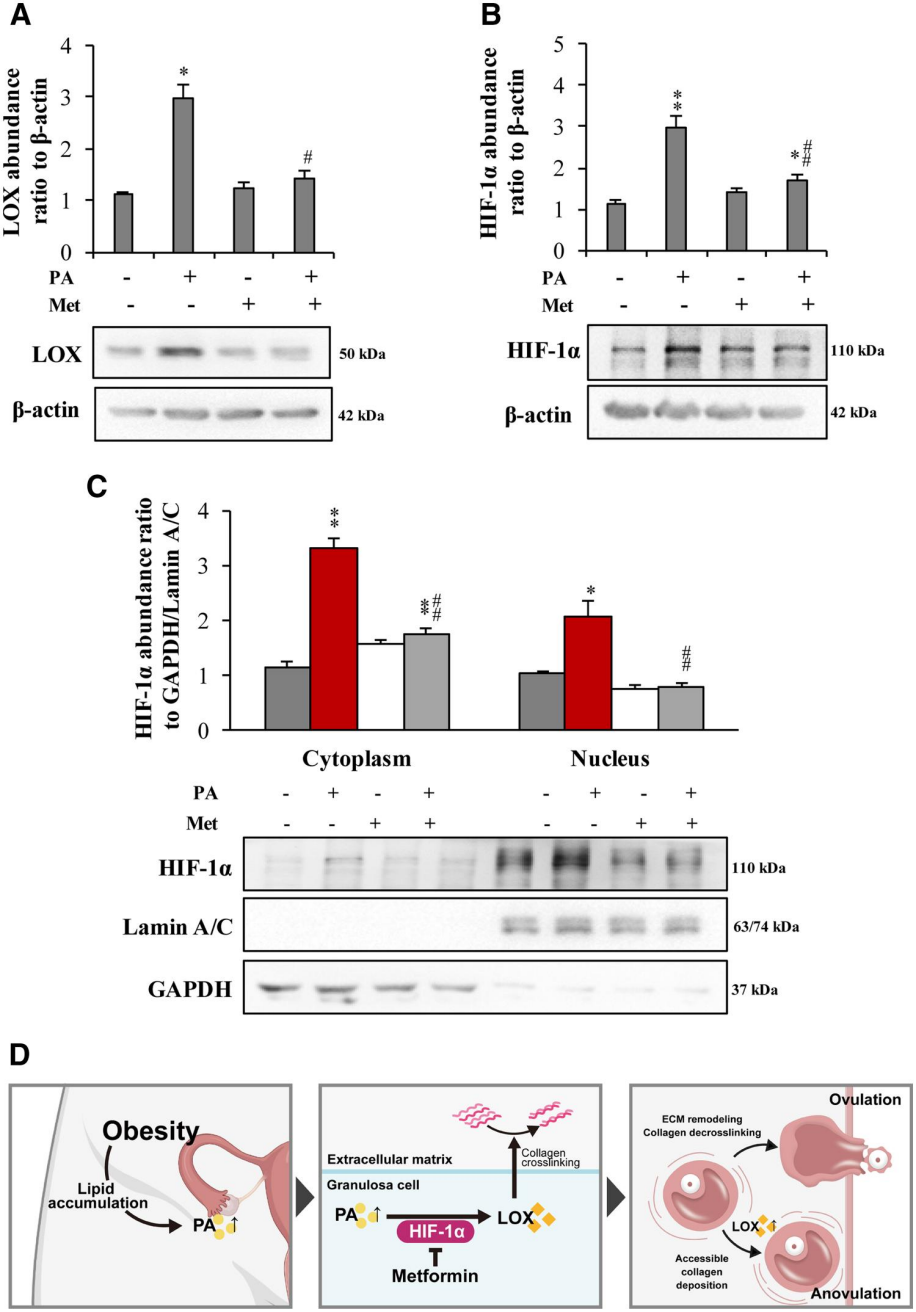
**Inhibition of palmitic acid-induced lysyl oxidase and hypoxia-inducible factor-1α expression by metformin in human primary granulosa cells *in vitro*.** (**A** and **B**) Effect of palmitic acid (PA) (300 μM, 24 h) on the protein abundance of LOX and HIF-1α in the presence and absence of metformin (Met, 1 mM, 24 h). (**C**) Effect of PA (300 μM, 24 h) on HIF-1α nuclear translocation in the presence or absence of metformin (1 mM, 24 h). The data are presented as the mean ± SEM from three or four experiments. Statistical analysis was performed with one-way ANOVA tests followed by Student–Newman–Keuls multiple comparisons tests. *P < 0.05, **P < 0.01 vs the control group; ^#^*P* < 0.05, ^##^*P* < 0.01 vs the PA-treated group. (**D**) Diagram illustrating the major findings of this study. HIF-1α: hypoxia-inducible factor-1α; LOX: lysyl oxidase.

Cotreatment of human granulosa cells with PA (300 μM, 24 h) and metformin (1 mM, 24 h) significantly attenuated the induction of LOX and HIF-1α expression and HIF-1α nuclear translocation induced by PA (Fig. 6A–C), indicating that metformin may improve ovulation through attenuation of LOX-mediated excessive collagen crosslinking in obese women.

## Discussion

This study demonstrated that PA in obesity induces LOX expression and excessive collagen deposition in the ovary, which may restrict follicle growth, impair ovulation, and lead to infertility. Metformin improved ovulatory function in individuals with obesity by attenuating LOX expression and excessive collagen deposition in the ovary (Fig. 6D). To our knowledge, this is the first study showing that metformin may be of therapeutic value for improving ovulation in individuals with obesity through its local effect on ECM remodelling in the ovary. Since our *in vitro* study of human granulosa cells showed that metformin could attenuate PA-induced LOX expression via attenuation of HIF-1α activation, we believe that the effects of metformin observed in this study are independent of its systemic effect on insulin sensitivity and chronic inflammation (Srinivasan *et al.*, 2006; Jing *et al.*, 2018).

In this study, we demonstrated that PA might stimulate LOX expression through the activation of HIF-1α in ovarian granulosa cells. Multiple hypoxia response elements (HREs) have been demonstrated to exist in the *LOX* promoter (Wong *et al.*, 2011; Wang *et al.*, 2017). Studies in tumour cells have shown that HIF-1α can mediate LOX expression by binding to HREs in the *LOX* promoter so that intensive ECM remodelling can occur to meet the demand for tumour cell invasion and metastasis (Wang *et al.*, 2017). We found that PA increased not only HIF-1α abundance but also its nuclear translocation in human granulosa cells. HIF-1α overexpression has been shown to be associated with collagen accumulation and fibrosis in adipose tissue (Li *et al.*, 2016) and oxidative stress induced by intracellular reactive oxygen species (ROS) has been demonstrated to occur through transcriptional, translational, and post-translational mechanisms (Movafagh *et al.*, 2015). Moreover, PA has been shown to induce ROS generation through alteration of mitochondrial oxidative metabolism (Harlow *et al.*, 2003) and modification of the compositions of cellular and mitochondrial membranes (Innis and Clandinin, 1981; Lemieux *et al.*, 2008). In addition to mediating HIF-1α activation by PA, ROS have also been shown to mediate PA-induced autophagy in endothelial cells of the human umbilical vein (Chen *et al.*, 2019). Since autophagy is involved in the regulation of both primordial follicular development and atresia (Zhou *et al.*, 2019), we speculate that PA may also affect follicular development through the induction of ROS-mediated autophagy.

Accumulating evidence indicates that obesity is a condition of chronic inflammation (Fernández-Sánchez *et al.*, 2011). Our previous study demonstrated that the inflammatory cytokine IL-1β can induce LOX expression in the ovarian granulosa cells of patients with PCOS (Zhang *et al.*, 2018). It has been reported that PA can cause chronic inflammation in peripheral tissues by activating inflammatory signalling pathways similar to those activated by bacterial endotoxin via Toll-like receptor 4 (Kochumon *et al.*, 2018; Korbecki and Bajdak-Rusinek, 2019). Thus, we speculate that elevated PA levels in individuals with obesity may also enhance LOX expression and collagen deposition in the ovary through activation of the inflammatory pathway. To this end, metformin may also improve ovulation in individuals with obesity through the amelioration of chronic inflammation in the ovary. This notion merits further investigation in the future.

Although elevated PA levels are a typical feature of obesity (Boden, 1997, 2008), the levels of several other saturated and polyunsaturated FFAs, such as stearic acid (SA) and arachidonic acid, are also increased in the blood in individuals with obesity (Lu *et al.*, 2003). Previous studies have shown that higher levels of both PA and SA in follicular fluid may have harmful effects on oocyte maturation and implantation in obese women (Mirabi *et al.*, 2017). Thus, increased levels of other FFAs may also contribute to the development of anovulation in obesity.

Collectively, our findings provide new insight into the mechanism of anovulation in obesity. We have shown that increased PA levels in individuals with obesity are a cause of LOX overexpression in the ovary, which can result in excessive collagen deposition, thus leading to impaired ovulation. Moreover, we found that these effects of PA could be ameliorated by metformin, which further justifies the therapeutic use of metformin in the treatment of ovulation disorders in individuals with obesity.

## Supplementary Material

hoae002_Supplementary_Data

## Data Availability

The datasets generated during and/or analyzed during the current study are available from the corresponding author upon reasonable request.
