# Supplementary material for: Attenuation of palmitic acid-induced lysyl oxidase overexpression in the ovary contributes to the improvement of ovulation in obesity by metformin
Source: Hum Reprod Open. 2024 Jan 10;2024(1):hoae002. doi: 10.1093/hropen/hoae002 (PMC10850847; doi:10.1093/hropen/hoae002)
Supplement: hoae002_Supplementary_Data [file hoae002_supplementary_data.docx]

**
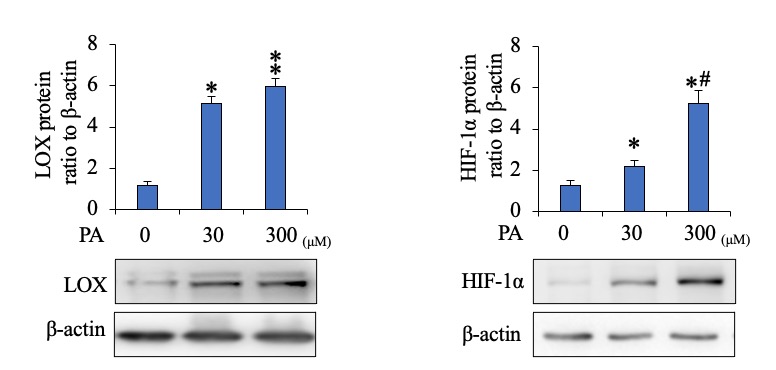
**

**Supplementary Figure S1** Induction of lysyl oxidase expression by palmitic acid through hypoxia-inducible factor-1α in KGN cells.

Concentration-dependent upregulation of LOX and HIF-1α expression by palmitic acid (PA) (0, 30, 300 μM, 24 hours) in KGN cells. Data are means ± SEM of three or four experiments. Statistical analysis was performed with one-way ANOVA tests followed by Student–Newman–Keuls multiple comparisons tests. **P* < 0.05, ***P* < 0.01, ****P* < 0.001 vs. control group; ^#^*P* < 0.05 vs. PA (30 μM)-treated group.

LOX: lysyl oxidase, HIF-1α: hypoxia-inducible factor-1α

**
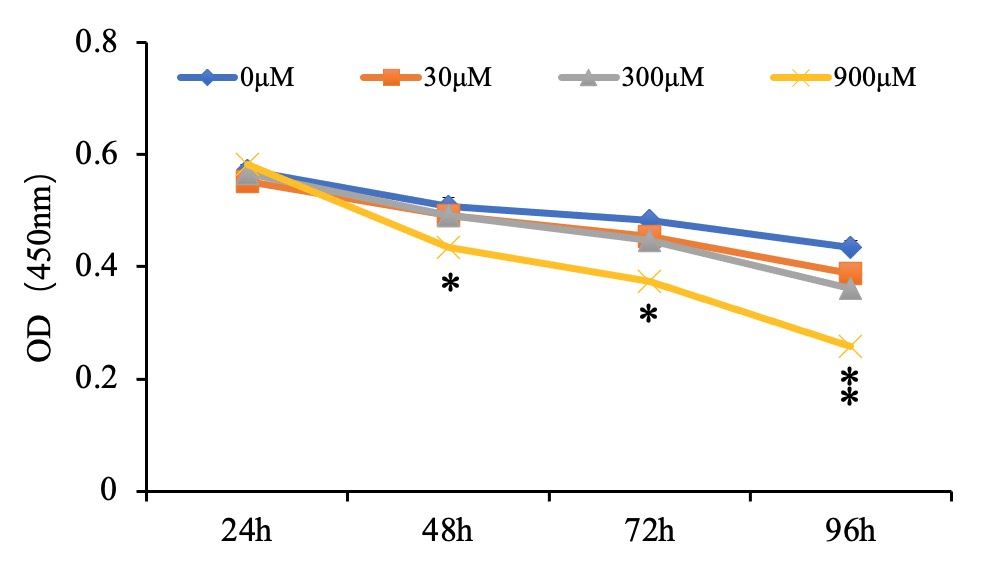
**

**Supplementary Figure S2** The cell viability of human granulosa cells treated with or without palmitic acid.

CCK-8 assay showing the effect of PA (0, 30, 300, 900 μM) on the cell viability of human granulosa cells. Cells was treated with or without PA for 24 , 48, 72 and 96 hours. The OD 450 nm value is proportional to the number of viable cells in the sample. Data are means ± SEM of three experiments. Statistical analysis was performed with one-way ANOVA tests followed by Student–Newman–Keuls multiple comparisons tests.**P* < 0.05, ***P* < 0.01 vs. control group (0 μM).

PA, palmitic acid; CCK-8, Cell counting kit-8.
